# Hybrid Conductive Hydrogels Reinforced by Core–Shell PANi@PAN Nanofibers for Resilient Electromechanical Stability at Subzero Temperatures

**DOI:** 10.3390/gels12050358

**Published:** 2026-04-24

**Authors:** Yuxuan Chen, Chubin He, Xiuru Xu

**Affiliations:** Key Laboratory of Optoelectronic Devices and Systems of Ministry of Education and Guangdong Province, College of Physics and Optoelectronic Engineering, Shenzhen University, Shenzhen 518000, China

**Keywords:** core–shell nanofibers, dual-conduction network, electron–ion hybrid conduction, anti-freezing hydrogels, electromechanical stability

## Abstract

Conductive hydrogels are attractive for flexible electronics, but their practical use is often limited by resistance drift during repeated deformation and performance degradation at low temperatures. Here, core–shell polyaniline-coated polyacrylonitrile (PANi@PAN) electrospun nanofibers were incorporated into a polyacrylamide/hydroxypropyl cellulose (PAM/HPC) hydrogel matrix to construct a hybrid conductive network. The PANi shell serves as an electronic pathway alongside ionic conduction in the hydrated polymer network, leading to markedly improved electromechanical stability. The resistance drift is about 11% after 2000 stretching–relaxation cycles at 0–100% strain, about 12 times lower than that of the nanofiber-free hydrogel. Stable electrical responses are maintained under large deformation, with a resistance drift as low as 3.3% over a strain range of 0–400%. The hydrogels show a conductivity of 0.32 S m^−1^ while retaining high stretchability (>600%). An ethylene glycol/water binary solvent is used to suppress ice formation and improve moisture retention, allowing stable electromechanical performance at −15 °C over 500 cycles. The hydrogel also adheres reliably to human skin (about 10.25 kPa) and functions as a conformal strain sensor without extra fixation.

## 1. Introduction

Flexible electronics have attracted extensive attention in recent years owing to their mechanical compliance and potential integration with soft systems such as wearable devices, biomedical platforms, and human–machine interfaces [1,2,3,4]. Among various material candidates, conductive hydrogels are particularly attractive because of their intrinsic softness, high water content, and tunable electrical properties, which enable intimate mechanical coupling with soft and deformable surfaces [5,6,7]. However, achieving stable electromechanical performance under repeated deformation and low-temperature environments remains a fundamental challenge for hydrogel-based conductive systems, largely due to the intrinsic instability of hydrated gel networks.

In most conductive hydrogels, electrical conduction mainly relies on ion transport within the hydrated polymer network. When the material undergoes repeated stretching and relaxation, the polymer chains gradually rearrange. This process can modify the ionic transport pathways, sometimes causing elongation, disruption, or redistribution of conductive channels. As a result, the resistance of the hydrogel often drifts during long-term cycling, which reduces signal reliability [8]. To improve electrical conductivity, conductive polymers such as poly(3,4-ethylenedioxythiophene): polystyrene sulfonate (PEDOT: PSS), polyaniline (PANi), and polypyrrole (PPy) have been incorporated into hydrogel matrices [9,10,11,12]. Although these strategies can increase conductivity, directly blending conductive polymers often leads to aggregation and heterogeneous dispersion [13,14]. The conductive pathways formed in this way may also become unstable during repeated deformation, meaning that improved conductivity does not always ensure long-term electromechanical stability [15,16]. To address these challenges, various strategies have been explored to incorporate conductive polymers into hydrogels, among which conductive nanofiber construction has emerged as a promising approach. Compared with conventional direct blending or in situ polymerization of conductive polymers within the hydrogel matrix, the use of core–shell structured nanofibers offer distinct advantages [17,18]. In this strategy, PANi is coated onto a preformed PAN nanofiber template, so that the conductive component is spatially confined to the fiber surface and forms a continuous, well-dispersed conductive shell. This architecture helps reduce the aggregation of conductive polymers, improves the uniformity of conductive pathways, and better preserves the mechanical flexibility and structural integrity of the hydrogel. Moreover, the core–shell configuration facilitates the formation of a stable electronic percolation network that can better withstand repeated mechanical deformation, which is difficult to achieve with randomly distributed conductive fillers or homogeneously polymerized conductive hydrogels [19]. Therefore, the rational design of core–shell PANi@PAN nanofibers provides an effective route to improve conductivity and long-term electromechanical stability simultaneously in soft conductive hydrogels. PANi conducts electricity through its π-conjugated backbone after protonation doping, enabling efficient electronic transport along the polymer chains. By incorporating PANi-coated nanofibers as a stable electronic framework, a hybrid electron–ion conduction network can be constructed to suppress resistance drift that commonly occurs in ionically conductive hydrogels under repeated deformation [20,21]. Furthermore, because high conductive polymer loading may compromise the optical transparency and stretchability of hydrogels, achieving stable electromechanical performance at relatively low conductive polymer loading remains an important challenge.

Another limitation of conventional hydrogels is their poor performance at subzero temperatures. Water crystallization reduces ion mobility and significantly weakens the elasticity of the gel network. To address this issue, binary solvent systems containing ethylene glycol or glycerol have been introduced to suppress ice formation through hydrogen-bond interactions with water molecules [22,23,24]. Nevertheless, the introduction of non-volatile solvents can alter gel-network interactions and may affect the balance between electrical performance, mechanical compliance, and interfacial adhesion. Meanwhile, reliable adhesion between hydrogels and external substrates is essential for stable signal acquisition in soft electronic applications, yet strong adhesion often conflicts with mechanical softness and reversible detachment [25,26,27]. Therefore, from a gel-network engineering perspective, constructing conductive hydrogels that simultaneously achieve electromechanical stability, stable operation at subzero temperatures, and reliable yet gentle adhesion remains challenging. Such low-temperature tolerance may be relevant for wearable devices used in cold-climate regions or winter outdoor environments, where low ambient temperatures can be part of daily use.

In this work, we develop a network-engineered conductive hydrogel by incorporating core–shell polyaniline-coated polyacrylonitrile (PANi@PAN) electrospun nanofibers into a polyacrylamide (PAM)/hydroxypropyl cellulose (HPC) matrix containing an ethylene glycol (EG)/water binary solvent system. The interconnected PANi shells form a mechanically tolerant electronic framework embedded within the hydrated polymer network, which cooperates with ionic transport to stabilize conductive pathways under deformation. Meanwhile, the EG–water solvent system regulates hydrogen-bond interactions, enhancing moisture retention and suppressing ice formation, while reversible intermolecular interactions contribute to stable yet gentle interfacial adhesion. As a result, the resulting hydrogel exhibits suppressed resistance drift under cyclic deformation, stable operation under subzero conditions, and reliable adhesion to diverse substrates. The reliable adhesion supports its potential in skin-interfaced and wearable sensing applications, while the stable subzero performance highlights its environmental adaptability for flexible electronics operating under cold conditions. This work highlights a structural design strategy for stabilizing conductive networks in soft hydrogels subjected to coupled mechanical deformation and low-temperature environments.

## 2. Results and Discussion

### 2.1. Structural Design and Morphology of PAM/HPC/PANi@PANnfs Hydrogels

**Figure 1:**
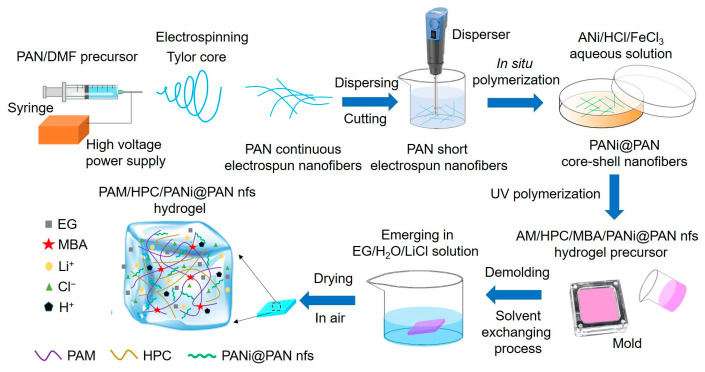
Schematic illustration of the structural design of the PAM/HPC/PANi@PANnfs hydrogel, including the formation of core–shell PANi@PAN nanofibers and their integration into the polymer network to support stabilized conductive pathways under mechanical deformation.

To construct a conductive hydrogel with enhanced electromechanical stability and environmental adaptability, a network-engineered architecture was designed, as schematically illustrated in Figure 1. In this design, core–shell PANi@PAN electrospun nanofibers are introduced as a mechanically tolerant conductive scaffold within a hydrated polymer matrix, aiming to stabilize conductive connectivity under deformation.

Electrospun PAN nanofibers were first prepared under an applied voltage of 15 kV to obtain uniform nanofibers. Subsequently, aniline (ANi) was polymerized in situ on the surface of the PAN nanofibers to form a conformal PANi shell, resulting in PANi@PAN core–shell nanofibers. In this configuration, the PAN core nanofiber provides mechanical flexibility and a nanofiber template, while the HCl-doped PANi shell serves as an electronically conductive layer. The core–shell architecture spatially confines PANi on the fiber surface, effectively mitigating aggregation and facilitating homogeneous dispersion within the hydrogel matrix, thereby building a continuous conductive network.

**Figure 2:**
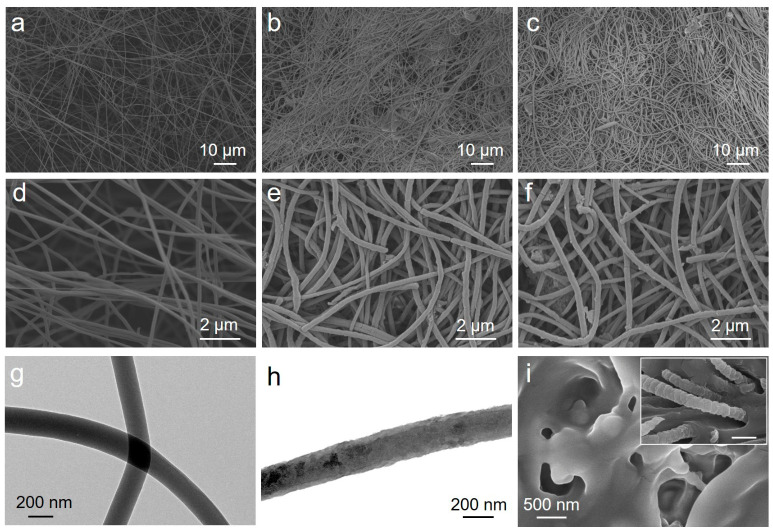
SEM images of (**a**) the as-electrospun PAN nanofibers, (**b**) PAN nanofibers after shearing treatment, and (**c**) the core–shell PANi@PAN nanofibers after in situ polymerization. (**d**–**f**) Higher-magnification SEM images of the samples shown in (**a**–**c**), respectively. TEM images of (**g**) the as-electrospun PAN nanofibers and (**h**) the core–shell PANi@PAN nanofibers, confirming the formation of the PANi shell. SEM image of (**i**) the pristine PAM/HPC hydrogel, with an inset showing the PAM/HPC/PANi@PANnfs hydrogel. The inset suggests the incorporation of nanofibers into the three-dimensional porous polymer network. The inset scale bar is 500 nm.

The morphology of the prepared samples was characterized by SEM and TEM, as shown in Figure 2. The as-electrospun PAN nanofibers exhibit relatively straight and smooth surfaces, with diameters mainly in the range of 150–250 nm (Figure 2a,d). After immersion in a water/ethanol mixed solution followed by shearing treatment, the PAN nanofibers become slightly thicker and more curved, with diameters of about 200–350 nm (Figure 2b,e), which may be associated with solvent-induced swelling during the liquid-phase treatment. Notably, the higher-magnification SEM image in Figure 2e reveals slit-like defects on some sheared PAN nanofibers, indicating that local damage was introduced during the shearing dispersion process. Because many fibers overlap and intertwine with each other in the SEM images, precise statistical analysis of fiber length is difficult. Based on the observable fiber segments, the post-shearing fiber length can only be roughly estimated to be on the order of tens to hundreds of micrometers, with some shorter fragments also present.

After in situ polymerization of aniline, the PANi@PAN nanofibers show a further increase in diameter to about 200–400 nm and more pronounced bending features (Figure 2c,f), consistent with the formation of a PANi coating on the fiber surface. A few truncated fiber ends and shorter fiber fragments can be observed after shearing and polymerization, suggesting that partial fragmentation occurred during the shearing treatment. TEM images further show that the as-electrospun PAN nanofibers have a smooth surface and a diameter of about 170 nm (Figure 2g), whereas the PANi@PAN nanofibers exhibit a distinct core–shell structure with a PANi shell thickness of approximately 20–40 nm (Figure 2h), further confirming the successful deposition of PANi on the PAN fiber surface. The pristine PAM/HPC hydrogel exhibits a typical three-dimensional porous structure (Figure 2i). As shown in the inset of Figure 2i, the PAM/HPC/PANi@PANnfs hydrogel still presents a porous network after nanofiber incorporation, and the incorporated nanofibers can be observed within the hydrogel matrix.

**Figure 3:**
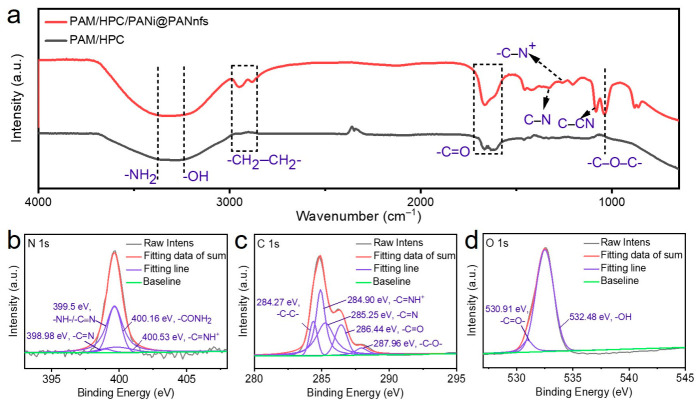
(**a**) FT-IR spectrum of the PAM/HPC/PANi@PANnfs hydrogel. High-resolution XPS spectra of the PAM/HPC/PANi@PANnfs hydrogel: (**b**) N 1s, (**c**) C 1s, and (**d**) O 1s.

Subsequently, FT-IR characterization was performed on the PAM/HPC/PANi@PANnfs hydrogel (Figure 3a). The band at 1649 cm^−1^ is assigned to the C=O stretching vibration of the amide groups in PAM, while the band at 1604 cm^−1^ is associated with the characteristic benzenoid/quinoid structure of PANi and overlapping amide-related vibrations. The broad bands at 3356 and 3297 cm^−1^ are attributed to the stretching vibrations of N–H and O–H groups in the hydrogel network. The band at 1033 cm^−1^ corresponds to the C–O–C stretching vibration of the HPC chains. The bands at 2942 and 2886 cm^−1^ are assigned to the asymmetric and symmetric stretching vibrations of aliphatic C–H bonds. In addition, the peaks at ~1203, 1255, and 1328 cm^−1^ are attributed to protonation-related C–N vibrations and aromatic amine stretching in PANi, indicating the formation of doped polyaniline in the composite hydrogel.

XPS characterization was carried out to analyze the chemical states of the PAM/HPC/PANi@PANnfs hydrogel (Figure 3b). The high-resolution N 1s spectrum can be deconvoluted into four characteristic components located at 398.98, 399.50, 400.16, and 400.53 eV, corresponding to imine nitrogen in PANi, amine-type nitrogen in PANi with possible contribution from PAN, amide nitrogen in PAM, and protonated nitrogen species in doped PANi, respectively. The C 1s spectrum in Figure shows characteristic peaks assigned to C–C/C=C, C–N/C–O, and C=O related chemical environments in the composite polymer network. As shown in Figure 3d, the O 1s spectrum can be fitted into two components associated with carbonyl oxygen and hydroxyl/ether oxygen species in the gel network. These results together confirm the successful incorporation of PANi@PAN nanofibers into the PAM/HPC hydrogel matrix.

### 2.2. Mechanical and Conductivity Properties of the PAM/HPC/PANi@PANnfs Hydrogels

**Figure 4:**
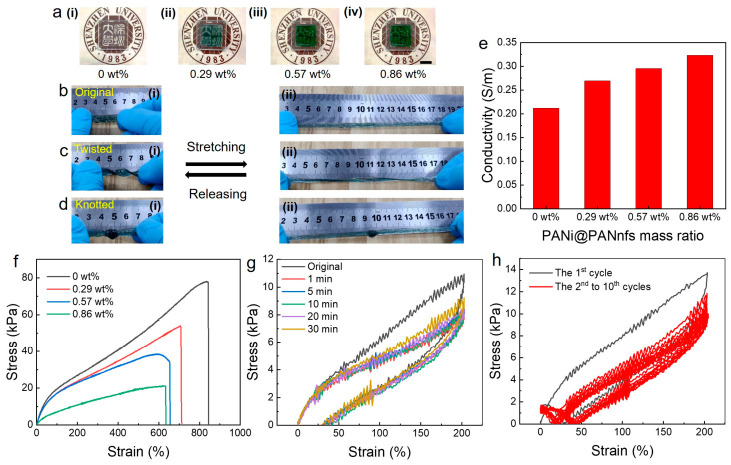
Mechanical and electrical properties of PAM/HPC/PANi@PANnfs hydrogels with different PANi@PANnfs concentrations. (**a**) Optical photographs of hydrogels containing 0 (**i**), 0.29 (**ii**), 0.57 (**iii**), and 0.86 wt% (**iv**) PANi@PANnfs of the PAM/HPC/PANi@PANnfs hydrogels (The scale bar is 2 cm). (**b**–**d**) Deformation behavior of the PAM/HPC/PANi@PANnfs hydrogel with 0.57 wt% PANi@PANnfs of the original (**b**), twisted (**c**), and knotted (**d**) state. For each state, the hydrogel is shown at 0% strain (**i**) and after stretching to over 400% strain (**ii**), demonstrating excellent mechanical resilience. (**e**) Electrical conductivity of PAM/HPC/PANi@PANnfs hydrogels as a function of the PANi@PANnfs concentrations. (**f**) Tensile stress–strain curves of hydrogels with different PANi@PANnfs concentrations. (**g**) Cyclic stress–strain curves under repeated deformation from 0 to 200% strain with different rest intervals of the PAM/HPC/PANi@PANnfs hydrogel with 0.57 wt% PANi@PANnfs. (**h**) Ten loading–unloading tensile cycles at 200% strain without relaxation time between cycles of the PAM/HPC/PANi@PANnfs hydrogel with 0.57 wt% PANi@PANnfs.

To study the mechanical and electrical properties of the PANi@PANnfs-reinforced hydrogel, the performance of the hydrogels was systematically investigated as a function of core–shell nanofiber loading (0, 0.29, 0.57 and 0.87 wt%). The optical appearance of the hydrogels gradually changes from transparent and colorless to green and semi-transparent with increasing PANi@PANnfs concentration, as shown in Figure 4a. This color transition is due to the increasing amount of PANi-coated nanofibers incorporated into the polymer matrix, confirming the successful introduction of conductive electrospun nanofibers into the hydrogel network. The deformation capability of the optimized hydrogel (0.57 wt% PANi@PANnfs) is illustrated in Figure 4b–d. The hydrogel maintains structural integrity under various deformation modes, including stretching from the initial state to strains exceeding 400% (Figure 4b(i,ii)), twisted deformation (Figure 4c(i,ii)), and knotted deformation (Figure 4d(i,ii)). During the testing, no visible cracks or structural failure were observed, indicating the excellent mechanical resilience and flexibility of the nanofiber-reinforced hydrogel.

The electrical conductivity increases as the PANi@PANnfs mass ratio rises from 0 to 0.86 wt%. At 0.86 wt%, the conductivity reaches 0.32 S m^−1^, which is about 1.5 times higher than that of the PAM/HPC hydrogel without PANi@PANnfs (Figure 4e). The TEM image in Figure 2h shows that the PANi shell thickness is approximately 20–40 nm, while the overall fiber diameter is around 240 nm. This observation suggests that the PANi shell amount for only part of the fiber cross-section. Even with this core–-shell naofibers structure, the conductivity of the hydrogel still increases after introducing PANi@PANnfs. Even with this relatively low PANi content, the conductivity of the hydrogel still increases after introducing PANi@PANnfs. The PANi shells formed on the nanofiber surface become conductive after HCl doping, where PANi exists in the protonated emeraldine salt state. Charge transport can then occur along the conjugated PANi chains. When the PANi-coated fibers are dispersed in the hydrogel matrix, contacts between neighboring fibers create multiple conductive junctions. These contacts provide electronic pathways across the hydrogel network and coexist with the ionic conduction of the PAM/HPC matrix.

The tensile stress–strain curves of hydrogels with different PANi@PANnfs contents are presented in Figure 4f. With increasing nanofiber loading, both the fracture strain and fracture stress gradually decrease. Two factors may contribute to this trend. First, PANi can partially absorb UV light during polymerization, which may slightly influence the effective crosslinking of the polymer network. Second, the presence of nanofibers locally restricts the mobility of polymer chains and reduces the overall deformability of the matrix. Even so, all samples remain highly stretchable, with fracture strains above 600%. Mechanical durability was further examined using cyclic tensile tests. As shown in Figure 4g, the hydrogel displays stable cyclic stress–strain curves during repeated deformation between 0 and 200% strain under different rest intervals. The loading–unloading curves in Figure 4h also show nearly identical responses over ten continuous stretching–releasing cycles at 200% strain without relaxation time, suggesting good mechanical resilience and structural stability.

### 2.3. Strain Sensing Performance and Electromechanical Stability of the PAM/HPC/PANi@PANnfs Hydrogels

**Figure 5:**
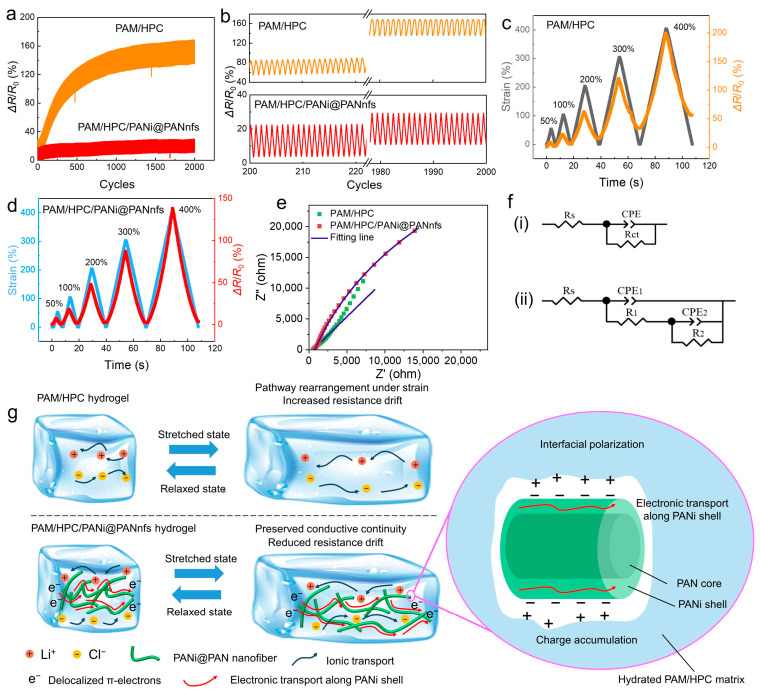
Electromechanical stability of PAM/HPC/PANi@PANnfs hydrogels. (**a**) Cyclic resistance response of hydrogels with (red line) and without PANi@PANnfs (orange line) during repeated tensile deformation between 0% and 100% strain for 2000 cycles. (**b**) Enlarged views of representative cycles extracted from (**a**), highlighting resistance drift behavior between the 200th–220th cycles and the 1980th–2000th cycles. (**c**,**d**) Relative resistance variation (Δ*R*/*R*_0_) of hydrogels without (**c**) and with (**d**) PANi@PANnfs under progressive tensile deformation (0 → 50% → 0 → 100% → 0 → 200% → 0 → 300% → 0 → 400%). (**e**) The electrochemical impedance spectroscopy (EIS) of the PAM/HPC hydrogel and the PAM/HPC/PANi@PANnfs hydrogel. (**f**) The equivalent circuit models fitted from the electrochemical impedance spectra of the hydrogel samples: (**i**) the PAM/HPC hydrogel and (**ii**) the PAM/HPC/PANi@PANnfs hydrogel. (**g**) Schematic illustration of the electron–ion hybrid conductive network, where PANi@PAN nanofibers form mechanically stable electronic pathways that cooperate with ionic conduction in the hydrogel matrix.

The electromechanical stability of conductive hydrogels is critically governed by the evolution of conductive pathways under repeated mechanical deformation. To evaluate the stabilization effect introduced by PANi@PAN core–shell nanofibers, cyclic tensile tests between 0% and 100% strain were performed for up to 2000 stretching–relaxation cycles on hydrogels with and without PANi@PANnfs. As shown in Figure 5a, the PAM/HPC hydrogel without PANi@PANnfs exhibits significant resistance accumulation during cyclic loading, with the baseline resistance gradually increasing to approximately 135% of its initial value after 2000 cycles. The enlarged views in Figure 5b further highlight the progressive drift of the resistance baseline when comparing early cycles (the 200th–220th cycles) with late cycles (the 1980th–2000th cycles). This behavior indicates the instability of ion-dominated conductive pathways with repeated mechanical deformation.

In contrast, the PANi@PANnfs-reinforced PAM/HPC hydrogel maintains a significantly better resistance drift performance than that of the PAM/HPC hydrogel. It remained below approximately 11% throughout the entire cycling test (Figure 5a), which is 1/12 of the resistance drift of the PAM/HPC hydrogel. The electromechanical stability is significantly improved by introducing the PANi@PANnfs into the PAM/HPC hydrogel, which indicates that the conductive network formed in the nanofiber-reinforced hydrogel is substantially more tolerant to cyclic deformation. The improvement becomes more evident under progressive tensile deformation (0 → 50% → 0 → 100% → 0 → 200% → 0 → 300% → 0 → 400%), as shown in Figure 5c,d. The fiber-free PAM/HPC hydrogel exhibits substantial resistance drift of 56.1% at higher strain levels (Figure 5c), indicating unstable conductive pathways under large deformation. By contrast, the PANi@PANnfs-reinforced hydrogel retains stable and reproducible resistance responses within a resistance drift of 3.3% across the entire strain range (Figure 5d), which is 1/17 of the resistance drift of the PAM/HPC hydrogel without PANi@PANnfs. Even at strains as high as 400%, the resistance signals remain clearly distinguishable and recoverable, demonstrating that the conductive network in the reinforced hydrogel can withstand large and repeated mechanical deformation.

The improved cyclic stability of the PANi@PANnfs-reinforced hydrogel is mainly related to the introduction of conductive nanofibers, which enable a combined ion–electron transport process. In the PAM/HPC hydrogel without nanofibers, electrical conduction mainly depends on the migration of mobile ions (Li^+^ and Cl^−^) in the hydrated polymer network. When the material is repeatedly stretched, the polymer chains gradually rearrange. As a result, the ionic transport pathways become longer and partially reorganized, which leads to a gradual increase in resistance. The PAM/HPC network also shows obvious mechanical hysteresis during repeated loading and unloading. This is due to the polymer’s intrinsic viscoelasticity and energy dissipation from friction between chain segments. Because of this hysteresis, the network cannot fully recover immediately after unloading. The temporary delay in structural recovery may cause a mismatch between the expanded ionic pathways and their original state, which contributes to resistance drift during rapid cycling.

After introducing PANi@PAN core–shell nanofibers, the situation becomes different. The introduced nanofibers provide an additional conductive framework that is less sensitive to polymer-chain rearrangement during deformation. Electrical pathways can therefore remain connected even when the hydrogel deforms. Interactions between the nanofibers and surrounding polymer chains may also help the network recover after deformation, which may help maintain stable electromechanical performance during repeated cycles.

### 2.4. Mechanistic Insight into the Hybrid Electron–Ion Conduction Network

Electrochemical impedance spectroscopy (EIS) was performed on the hydrogel samples (Figure 5e). Compared with the pristine PAM/HPC hydrogel, the PAM/HPC/PANi@PANnfs hydrogel exhibits a clearly different impedance response, indicating that the introduction of PANi@PAN nanofibers changes the overall charge-transport behavior of the system and is consistent with altered interfacial characteristics within the composite. The difference in the Nyquist plots suggests that the conductive behavior of the composite hydrogel cannot be fully described by a simple ion-conduction process alone. Instead, it is more reasonably understood as the combined result of ionic transport, electronic transport, and interfacial effects associated with the PANi@PAN nanofiber phase [13,28,29,30,31]. The impedance spectra were further analyzed using the equivalent circuit models shown in Figure 5f. In these models, *R*_s_ represents the bulk resistance of the system, including the intrinsic resistance of the hydrogel and contact-related resistance, while the constant phase element (CPE) is used to describe the non-ideal capacitive behavior of heterogeneous interfaces. For the PAM/HPC/PANi@PANnfs hydrogel, the additional fitted resistance/capacitance elements indicate a more complex transport process than that of the pristine PAM/HPC hydrogel. This difference is consistent with the introduction of an additional conductive phase and with possible interfacial polarization arising from the coexistence of the ion-conducting hydrogel matrix and the PANi-coated nanofiber network. Therefore, the EIS results support a coupled transport picture rather than a simple additive model of independent ionic and electronic conduction [32,33].

In the pristine PAM/HPC hydrogel, charge transport mainly relies on the migration of mobile ions in the water/ethylene glycol-containing polymer network [34]. During repeated stretching, deformation-induced rearrangement of the polymer chains and solvent-rich ionic pathways can alter conductive continuity, which contributes to resistance hysteresis and progressive resistance drift during cyclic loading. After incorporation of PANi@PAN nanofibers, an additional electronically conductive pathway is introduced into the hydrogel. PANi is a conjugated polymer capable of electronic conduction, and the XPS results suggest protonated PANi species consistent with a conductive doped state under the present experimental conditions. Thus, electron transport along the PANi shell is likely to contribute to the overall conductivity of the composite system, while the hydrated PAM/HPC matrix continues to provide ionic conduction. As a result, a hybrid electron–ion conductive network is proposed for the PAM/HPC/PANi@PANnfs hydrogel. Under the present experimental conditions, the PANi shell is discussed here as a protonated conductive phase that contributes to electronic transport (Figure 5g).

At the interface between the PANi@PAN nanofibers and the hydrated hydrogel matrix, the transport behavior may be more complex because the coexistence of ionic and electronic pathways can give rise to heterogeneous interfacial effects. This interface can therefore be viewed as a heterogeneous transport region, where differences in transport pathway and local charge mobility may lead to charge accumulation and possible interfacial polarization. Such interfacial effects are likely to influence the impedance response of the composite system and may also affect its electromechanical behavior under deformation. The present results do not directly resolve the microscopic details of carrier exchange at the hydrogel/fiber interface, but they support a hybrid transport picture involving ionic conduction, electronic conduction, and interfacial polarization. This hybrid conductive architecture is beneficial for electromechanical stability. During repeated tensile deformation, the ionic conductive pathways in the hydrogel matrix may still undergo elongation, local disruption, and reconstruction. However, the PANi@PAN nanofibers serve as a mechanically compliant conductive framework embedded in the soft matrix. Because of their fibrous geometry and flexibility, these nanofibers can accommodate deformation through bending, sliding, and local rearrangement, thereby helping preserve conductive connectivity during cyclic stretching [35,36]. This structural role is consistent with the significantly reduced resistance drift observed for the PAM/HPC/PANi@PANnfs hydrogel compared with the pristine PAM/HPC hydrogel. Unlike the pristine hydrogel that depends primarily on ion migration, the PAM/HPC/PANi@PANnfs hydrogel exhibits a more stable hybrid conductive architecture. Its conductive behavior can be understood as the combined result of ionic conduction in the hydrated polymer network, electronic conduction along the PANi-coated nanofibers, and interfacial polarization between these two phases. This combined transport mechanism is consistent with the improved electromechanical stability and the reduced resistance drift of the composite hydrogel during long-term cyclic stretching.

### 2.5. Solvent-Mediated Anti-Freezing and Moisture-Retention Behavior

**Figure 6:**
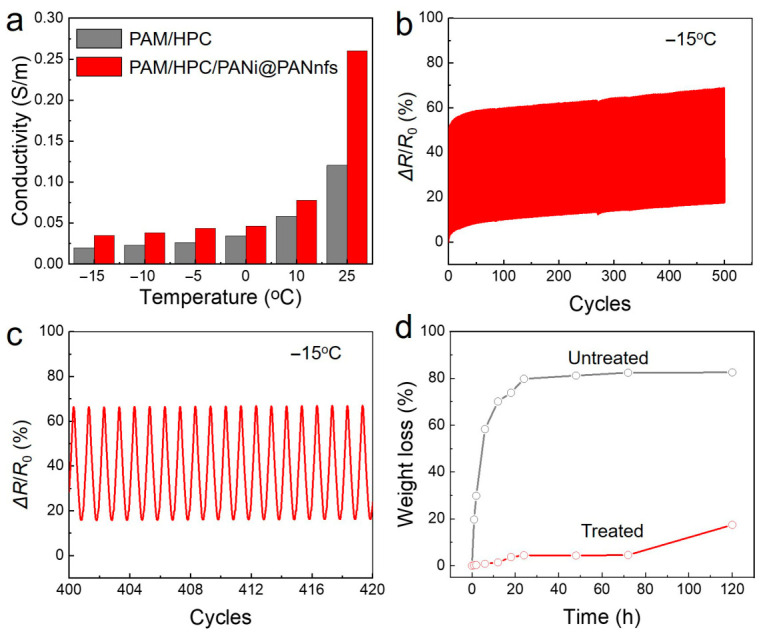
Low-temperature and moisture-retention performance of PAM/HPC/PANi@ PANnfs hydrogels after binary solvent treatment. (**a**) Electrical conductivity of hydrogels measured from −15 °C to 25 °C. (**b**) Cyclic resistance response under repeated tensile deformation between 0% and 100% strain at −15 °C for 500 cycles. (**c**) Enlarged view of the 400th–420th cycles extracted from (**b**). (**d**) Mass loss of untreated and solvent-treated hydrogels measured at 25 °C and 60% relative humidity.

Traditional hydrogels usually fail to maintain their electrical and mechanical performance at subzero temperatures because freezing of the water-rich domains stiffens the polymer network and restricts ion transport. Water evaporation under ambient conditions can also cause shrinkage of the gel matrix and deterioration of its electrical and mechanical properties. Replacing part of the water with a non-volatile solvent is an effective way to improve both low-temperature tolerance and environmental stability. Such solvent molecules contain abundant hydroxyl groups and can form hydrogen bonds with both water molecules and the polymer framework, thereby suppressing ice formation and reducing solvent evaporation. The temperature-dependent conductivity of the hydrogels was measured from 25 °C to −15 °C, as shown in Figure 6a. For both the PAM/HPC hydrogel and the PAM/HPC/PANi@PANnfs hydrogel, the electrical conductivity gradually decreases with decreasing temperature, which is mainly attributed to the reduced mobility of ions in the hydrogel network at low temperatures. However, the conductivity of the PAM/HPC/PANi@PANnfs hydrogel remains consistently higher than that of the pristine PAM/HPC hydrogel over the entire tested temperature range. This result indicates that the PANi@PANnfs network provides an additional conductive pathway in the hydrogel and helps maintain a higher overall conductivity when ionic transport becomes less efficient at low temperatures.

The low-temperature electromechanical stability of the PAM/HPC/PANi@PANnfs hydrogel was further evaluated by cyclic tensile tests at −15 °C for 500 loading–unloading cycles from 0 to 100% strain, as shown in Figure 6b. The resistance drift remains below 17% over 500 cycles, which is only about 54% higher than that at room temperature. The response from the 400th to 420th cycles is shown in Figure 6c, where the signal output remains stable without obvious deterioration, further confirming the reliable electromechanical response of the hydrogel under repeated deformation at subzero temperature. Moisture retention was studied by monitoring mass loss for 120 h at 25 °C and 60% relative humidity (Figure 6d). The untreated hydrogel loses water rapidly, with mass loss exceeding 58% within 6 h and reaching more than 80% after 24 h. After solvent exchange, the behavior changes markedly. The solvent-treated hydrogel shows much slower mass loss, remaining below 4.5% after 72 h and about 17.5% after 120 h. The presence of EG in the solvent phase reduces evaporation and helps maintain hydration of the polymer network during storage. These results show that the binary solvent treatment improves both low-temperature tolerance and moisture-retention behavior of the hydrogel.

### 2.6. Interfacial Adhesion and Skin-Conformal Behavior

**Figure 7:**
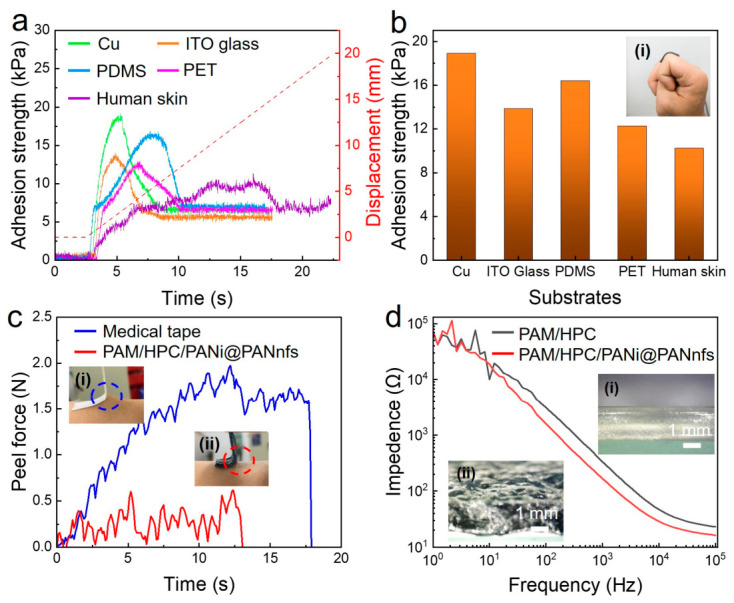
Interfacial adhesion and skin–electrode contact characteristics of PAM/HPC/PANi@PANnfs hydrogels. (**a**) Adhesion force–displacement curves of hydrogels on different substrates. (**b**) Maximum adhesion strength on various materials; inset: hydrogel attached to a finger joint. (**c**) Peel force on human skin for medical tape (**i**) and hydrogel (**ii**). (**d**) Skin–electrode impedance as a function of frequency for PAM/HPC and PANi@PANnfs hydrogels; insets: optical images of hydrogel surfaces without (**i**) and with (**ii**) PANi@PANnfs.

Stable interfacial contact is important for maintaining reliable electrical signals in soft conductive systems. Weak adhesion between a hydrogel and a substrate can lead to interfacial sliding and unstable contact resistance. The adhesion behavior of the PAM/HPC/PANi@PANnfs hydrogel was examined using 90° peel tests on different substrates (Figure 7a,b). The hydrogel shows measurable adhesion on metallic, polymeric, and biological surfaces. As shown in Figure 7a, the force–displacement curves display similar adhesion responses on the tested substrates.

The maximum adhesion strengths (Figure 7b) reach 18.91 kPa on Cu, 13.88 kPa on ITO glass, 16.42 kPa on PDMS, and 12.28 kPa on PET. Particularly, when the hydrogel is attached to human skin, its adhesion strength is about 10.25 kPa. This indicates that the hydrogel can maintain reliable interfacial adhesion, which means stable resistance signals during joint movements, like finger bending, caused by local skin deformations. This stable skin interface may result from reversible hydrogen bonding between the hydrogel surface and the skin. The surface microstructure also promotes conformal contact between the two surfaces. Close interfacial contact increases van der Waals interactions, which may help maintain stable adhesion. The hydrogel adheres to the skin without the need for additional adhesive tapes or fixation layers. Peel force measurements were used to evaluate detachment behavior on skin, with a conventional medical tape used for comparison. In Figure 7c, the medical tape shows a much higher maximum peel force of about 2.0 N, roughly four times that of the PAM/HPC/PANi@PANnfs hydrogel. As shown in Figure 7c(i), removal of the medical tape lifts the skin together with the tape at an angle of about 45°. In contrast, when the hydrogel is detached, the skin surface remains nearly perpendicular to the hydrogel (close to 90°, Figure 7c(ii)). A smaller peeling angle usually corresponds to higher local stress applied to the skin during detachment, which may lead to greater discomfort and even skin damage. In comparison, the hydrogel can be peeled off from the skin easily with minimal discomfort. This mild detachment behavior reduces the risk of pain or potential skin damage associated with medical tapes and other adhesive-assisted wearable devices. Such characteristics are beneficial for sensitive or fragile skin areas and suggest that the hydrogel can serve as a comfortable wearable sensor and even a soft interface between wearable sensors and the skin. Skin–electrode impedance was measured from 0.1 Hz to 10 kHz (Figure 7d). The PANi@PANnfs-reinforced hydrogel shows lower impedance than the PAM/HPC hydrogel in the mid- and high-frequency ranges. At 100 Hz, the impedance decreases from 3.2 kΩ to 1.6 kΩ. At 10 kHz, the value decreases from 47.0 Ω to 28.8 Ω. The lower skin–electrode impedance may be attributed to the combined effects of the conductive nanofiber network and the conformal contact of the hydrogel surface with skin.

The presence of PANi@PAN nanofibers also slightly changes the gel structure during polymerization. Optical micrographs in Figure 7d (insets) show that the reinforced hydrogel surface is rougher than that of the PAM/HPC hydrogel. The resulting micro-texture increases the effective contact area at the hydrogel–skin interface. Reversible interactions between functional groups in the polymer network, including amide groups in PAM, hydroxyl groups in HPC and EG, and amine groups in PANi, further contribute to interfacial adhesion. These features allow the hydrogel to maintain stable but reversible contact with skin during repeated deformation.

The key performance parameters of the PANi@PANnfs-reinforced hydrogel were compared with those of representative conductive hydrogels and organohydrogels reported in recent studies, as summarized in Table 1. Some previously reported systems show higher stretchability or have been evaluated at lower temperatures, but long-term resistance drift during repeated loading is often not explicitly quantified. Our hydrogel shows a good balance of properties, including a conductivity of 0.32 S m^−1^, a fracture strain above 600%, stable operation at −15 °C, low mass loss of less than 17.5% after 120 h, and reliable adhesion to human skin of 10.25 kPa. The resistance drift remains below 11% during 2000 tensile cycles at 100% strain. Compared with the fiber-free counterpart, the much lower resistance drift further indicates that the core–shell PANi@PAN nanofiber network helps improve long-term electromechanical stability. These results indicate that the present hydrogel compares favorably with previously reported hydrogel-based strain sensors in terms of cyclic stability, low-temperature tolerance, moisture retention, and interfacial conformability.

### 2.7. Strain-Sensing Monitoring in Human Motion Monitoring

**Figure 8:**
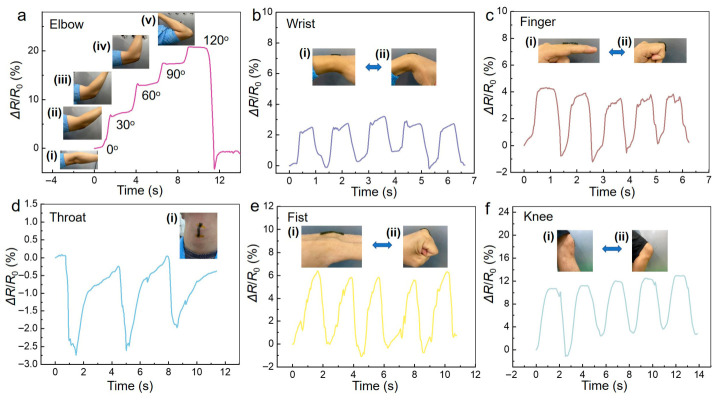
Strain-sensing responses of the PAM/HPC/PANi@PANnfs hydrogel during different human motions. (**a**) Elbow flexion at different angles: 0° (**i**), 30° (**ii**), 60° (**iii**), 90° (**iv**), and 120° (**v**). (**b**) Continuous wrist motion during extension (**i**) and flexion (**ii**). (**c**) Finger motion during extension (**i**) and flexion (**ii**). (**d**) Detection of swallowing motion. (**e**) Fist motion during releasing (**i**) and clenching (**ii**). (**f**) Knee motion during extension (**i**) and flexion (**ii**). Insets display the corresponding deformation states of the hydrogel during measurement.

The strain response of the hydrogel was examined through several human motion tests (Figure 8). The PAM/HPC/PANi@PANnfs hydrogel can be directly attached to the skin because of its mechanical compliance and interfacial adhesion.

The hydrogel therefore deforms together with the underlying skin during body movement. When placed on the elbow, the resistance increases as the elbow bends from 0° to 120° (Figure 8a). Wrist and finger flexion–extension motions produce repeatable ΔR/R_0_ signals (Figure 8b,c). The hydrogel shows an obvious resistance response during swallowing (Figure 8d). Cyclic clenching and releasing of the fist, as well as knee flexion–extension motions, produce distinct resistance signals (Figure 8e,f). The resistance signals remain stable during repeated movements, demonstrating reliable electrical responses under different deformation modes.

## 3. Conclusions

In this work, a hybrid conductive hydrogel with improved electromechanical stability and low-temperature tolerance was developed by integrating core–shell PANi@PAN electrospun nanofibers into a PAM/HPC matrix. The core–shell architecture enables the formation of a continuous electronic network that cooperates with ionic conduction in the hydrated polymer matrix, thereby stabilizing conductive pathways under repeated deformation. As a result, the nanofiber-reinforced hydrogel shows significantly reduced resistance drift, remaining below 11% over 2000 cycles at 100% strain compared with the fiber-free counterpart. The introduction of an ethylene glycol/water binary solvent further suppresses ice formation and improves moisture retention, allowing stable electromechanical performance at −15 °C, which was the lowest temperature evaluated in this work. In addition, the hydrogel exhibits gentle yet stable adhesion to human skin without the need for extra fixation. These results demonstrate that combining nanofiber-reinforced hybrid conduction with solvent regulation is an effective strategy for developing mechanically robust, environmentally adaptable, and skin-conformal conductive hydrogels for flexible and wearable electronics.

## 4. Materials and Methods

### 4.1. Materials

Acrylamide (AM, ≥99%), hydroxypropyl cellulose (HPC), N,N′-methylene bis(acrylamide) (MBA, ≥99%), lithium chloride (LiCl, ≥99%), α-ketoglutaric acid (KGA, ≥99%), polyacrylonitrile (PAN, Mw ≈ 150,000), aniline (ANi, ≥99.5%), iron(III) chloride hexahydrate (FeCl_3_·6H_2_O, ≥98%), ethylene glycol (EG, ≥99%), ethanol (≥99.5%), N,N-dimethylformamide (DMF, ≥99.5%), and hydrochloric acid (HCl, 37 wt% aqueous solution) were purchased from Aladdin Inc. (Shanghai, China). All reagents were used as received without further purification. Deionized water was prepared using a laboratory purification system.

### 4.2. Preparation of PANi@PAN Core–Shell Nanofibers (PANi@PANnfs)

Electrospun PAN nanofibrous membranes were first prepared by electrospinning. Specifically, an 8 wt% PAN solution in *N,N*-dimethylformamide (DMF) was used as the precursor solution. The solution was electrospun at an applied voltage of 10 kV with a tip-to-collector distance of 12 cm and a feeding rate of 1.0 mL h^−1^. Electrospinning was carried out at a relative humidity of 40–50% using plate collector to obtain PAN nanofibrous membranes. The obtained PAN nanofibrous membrane was carefully peeled off from the collector and cut into small fragments of approximately 3 mm × 3 mm. Approximately 0.05 g of the PAN membrane fragments was then immersed in 20 mL of a DI water/ethanol mixed solution (volume ratio = 1:1) and subjected to high-speed shearing using a disperser (T10 basic, IKA, Staufen, Germany) to obtain a uniform PAN nanofiber suspension.

Aniline (ANi, 400 μL) was added to the suspension under magnetic stirring and allowed to adsorb onto the fiber surfaces for 30 min at room temperature. Subsequently, 20 mL of an aqueous solution containing iron(III) chloride hexahydrate (FeCl_3_·6H_2_O, 0.2 mol·L^−1^) and hydrochloric acid (HCl, 1 mol·L^−1^) was slowly introduced to initiate oxidative polymerization. To polymerize ANi monomer as PANi shell on the surfaces of the PAN nanofibers, we heated the mixture at 80 °C with continuous stirring. After 45 min, green PANi@PAN core–shell nanofibers were successfully obtained. To remove any potential residual monomers, we alternately applied centrifugation and filtration to wash the resulting green nanofibers with ethanol. Finally, we dispersed green PANi@PANnfs in 20 mL of ethanol for further use [42].

### 4.3. Preparation of PAM/HPC/PANi@PANnfs Hydrogel

PANi@PANnfs were collected from the ethanol dispersion by centrifugation and dried at 60 °C for 12 h. The dried nanofibers were redispersed in 5 mL of deionized water by ultrasonication for 10 min to obtain a uniform suspension. AM monomer (1.25 g), HPC (0.05 g), MBA (0.001 g), and 0.1 mol·L^−1^ HCl aqueous solution (0.5 mL) were added to the suspension and stirred at room temperature for 14 h to obtain a homogeneous mixture. The content of PANi@PANnfs was adjusted according to the desired formulation (0–0.86 wt%). α-Ketoglutaric acid (KGA, 0.03 g) was added as the photoinitiator, and the mixture was degassed for 5 min to remove trapped air bubbles. The precursor solution was poured into a mold (45 mm × 45 mm × 2 mm) and polymerized under UV light (365 nm, 15 mW·cm^−2^) for 10 min.

The polymerized hydrogel was immersed in a binary mixture of deionized water and ethylene glycol (H_2_O:EG = 1:2, *v*/*v*) with 0.1 mol·L^−1^ HCl and 5 wt% LiCl for 2. Part of the water in the polymer network was replaced by ethylene glycol during the solvent exchange process. In the original hydrogel, hydrogen bonds between the polymer chains and surrounding solvent are mainly associated with water molecules. After solvent exchange, ethylene glycol can interact with the polymer chains through its hydroxyl groups, forming similar hydrogen-bond interactions within the network.

### 4.4. Characterization

All measurements were repeated three times to ensure reproducibility.

#### 4.4.1. Mechanical Testing

Mechanical tensile tests were conducted on a universal testing machine (CTM2010, Xieqiang Instruments, Shanghai, China). The hydrogels were cut into 20 mm × 7 mm × 2 mm pieces.

#### 4.4.2. Electromechanical Measurements

Electromechanical measurements were carried out on the same tensile testing machine with a simultaneous resistance meter (34465A, Keithley, Solon, OH, USA). The electrical conductivity (*σ*) was calculated according to:

(1) $$\sigma =\frac{l}{R\;\times \;A}$$

where *R* is the measured resistance, *l* is the sample length, and *A* is the cross-sectional area (width × thickness).

#### 4.4.3. Skin–Electrode Impedance Measurement

Skin–electrode impedance was measured using an electrochemical workstation (CHI660E, CH Instruments, Shanghai, China). Two hydrogel electrodes (20 mm × 20 mm × 2 mm) were placed on the wrist with a center-to-center spacing of 40 mm. Electrochemical impedance spectroscopy (EIS) measurements were performed over a frequency range of 0.1 Hz to 10 kHz under ambient conditions.

#### 4.4.4. Adhesion Test

Adhesion properties were evaluated via a 90° peel test also using the CTM2010 universal testing machine. Hydrogel patches (13.5 mm × 13.5 mm) were pressed onto various substrates under a preload of 1 N for 2.5 s. The peel test was conducted at a constant speed of 1 mm s^−1^ until detachment occurred. The maximum peel force was recorded for analysis.

#### 4.4.5. Anti-Freezing Test

For low-temperature electromechanical evaluation, hydrogel specimens (20 mm × 7 mm × 2 mm) were mounted on a motorized tensile stage inside a temperature-controlled chamber (FQY/GDJS-800). Cyclic tensile tests were performed at −15 °C with a strain rate of 5 mm s^−1^. Resistance variation during deformation was recorded simultaneously using a digital multimeter (DMM6500, 6½-Digit, Keithley, Solon, OH, USA).

## Figures and Tables

**Table 1:** Comparison of key performance parameters of this work with representative conductive hydrogels and organohydrogels reported in recent studies.

	Electrical Conductivity (S·m^−1^)	Resistance Drift	Maximum Working Strain (%)	Tested Temperature (°C)	Moisturizing Properties	Adhesion Strength (kPa)
PAAm, PVA, Mxene, borax, EG, H_2_O [37]	0.04	—	350	−40	weight-retention up to 8 days	—
PAAm, κ-carrageenan, xylitol [38]	—	—	700	−38	mass loss 28.3–33.6% after 48 h	—
PAA, gelatin, TA, AlCl_3_, H_2_O, glycerol [39]	0.31	no significant attenuation during 100 cycles of 100% strain tensile	1250	−14	remained moist during the 28 days storage	26.8 kPa
PVP, P(AA-co-AAm), H_2_O, DMSO [40]	—	—	1554	−40	mass retention > 83% after 7 days	44 kPa
P(AAm-co-GG)@AMH, NaCl, EG, H_2_O [41]	0.0468	—	717	−24	5.8% weight loss after 120 h	—
This Work	0.32	<11% during 2000 cycles of 100% strain tensile	>600	−15	<17.5% mass loss after 120 h	18.91 kPa (to copper surface); 10.25 kPa (to human skin)

PAAm: polyacrylamide, PVA: polyvinyl alcohol, EG: ethylene glycol, PAA: polyacrylic acid, TA: tannic acid, PVP: polyvinylpyrrolidone, DMSO: dimethyl sulfoxide, GG: gellan gum, AMH: 2-Amino-1-methyl-5H-imidazol-4-one.

## Data Availability

The raw data supporting the conclusions of this article will be made available by the authors upon request.
